# Supplementary material for: SOX2 regulates acinar cell development in the salivary gland
Source: eLife. 2017 Jun 17;6:e26620. doi: 10.7554/eLife.26620 (PMC5498133; doi:10.7554/eLife.26620)
Supplement: Figure 2—source data 1. — qPCR analysis of E16.5 Krt14CreERT2; Sox2fl/fl and wild-type (WT) SMG+SLG for genes involved in acinar differentiation, ductal differentiation and innervation, with expression normalised to Rsp29 and the WT. n = 3 embryos per genotype. s.d. = standard deviation. DOI: http://dx.doi.org/10.7554/eLife.26620.005 [file elife-26620-fig2-data1.docx]

**Figure 2 – source data 1.** Source data relating to Figure 2C. qPCR analysis of E16.5 *Krt14^CreERT2^; Sox2^fl/fl^* and wild-type (WT) SMG+SLG for genes involved in acinar differentiation, ductal differentiation and innervation, with expression normalised to *Rsp29* and the WT. n=3 embryos per genotype. s.d. = standard deviation.

| **Gene** | **WT** | s.d. | ***Krt14^CreERT2^;Sox2^fl/fl^*** | s.d. |
| --- | --- | --- | --- | --- |
| *Cdh1* | 1.00 | 0.09 | 1.00 | 0.07 |
| *Fgf10* | 1.00 | 0.15 | 2.77 | 2.05 |
| *Sox2* | 1.00 | 0.57 | 0.45 | 0.37 |
| *Krt5* | 1.00 | 0.64 | 0.47 | 0.07 |
| *Aqp5* | 1.00 | 0.38 | 0.22 | 0.03 |
| *Chrm3* | 1.00 | 0.83 | 0.12 | 0.12 |
| *Mist1* | 1.00 | 1.02 | 0.29 | 0.23 |
| *Nrtn* | 1.00 | 0.15 | 0.52 | 0.31 |
| *Sox10* | 1.00 | 0.01 | 0.12 | 0.10 |
| *Muc19* | 1.00 | 1.00 | 0.01 | 0.00 |
| *Atoh1* | 1.00 | 1.67 | 0.01 | 0.04 |
| *Dccp1* | 1.00 | 0.79 | 0.16 | 0.01 |
| *Krt7* | 1.00 | 0.35 | 1.17 | 0.07 |
| *Krt15* | 1.00 | 0.67 | 1.13 | 0.52 |
| *Krt19* | 1.00 | 0.34 | 0.93 | 0.20 |
| *Egfr* | 1.00 | 0.25 | 5.06 | 1.37 |
| *Aqp3* | 1.00 | 0.45 | 5.17 | 2.13 |
| *Tubb3* | 1.00 | 0.68 | 1.68 | 0.42 |
| *Vip* | 1.00 | 0.54 | 1.07 | 0.50 |
| *Vacht* | 1.00 | 0.59 | 4.04 | 0.61 |
| *Chrm1* | 1.00 | 0.18 | 2.75 | 0.72 |
