# Supplementary material for: SOX2 regulates acinar cell development in the salivary gland
Source: eLife. 2017 Jun 17;6:e26620. doi: 10.7554/eLife.26620 (PMC5498133; doi:10.7554/eLife.26620)
Supplement: Figure 2—source data 2. — Quantification of cells expressing acinar or ductal markers, cleaved caspase-3 or Ki67 in acini of E16.5 in Krt14CreERT2; Sox2fl/fl and wild-type (WT). n = 2–4 glands/genotype and cells were counted in 3–4 acini/gland. s.d. = standard deviation. DOI: http://dx.doi.org/10.7554/eLife.26620.006 [file elife-26620-fig2-data2.docx]

**Figure 2 - source data 2.** Source data relating to Figure 2D. Quantification of cells expressing acinar or ductal markers, cleaved caspase-3 or Ki67 in acini of E16.5 in *Krt14^CreERT2^; Sox2^fl/fl^* and wild-type (WT). n = 2-4 glands/genotype and cells were counted in 3-4 acini/gland. s.d. = standard deviation.

| **Gene** | **WT** | s.d. | ***Krt14^CreERT2^;Sox2^fl/fl^*** | s.d. |
| --- | --- | --- | --- | --- |
| SOX10+ | 14.75 | 5.73 | 0.38 | 0.74 |
| AQP5+ | 10.75 | 3.45 | 1.38 | 1.19 |
| MIST1+ | 13.63 | 4.27 | 0.00 | 0.00 |
| KRT5+ | 16.33 | 3.56 | 4.17 | 1.17 |
| KRT19+ | 21.17 | 2.56 | 22.17 | 4.36 |
| CASP3+ | 0.50 | 0.76 | 4.50 | 1.85 |
| Ki67+ | 20.38 | 4.96 | 6.25 | 2.66 |
