# Supplementary material for: SOX2 regulates acinar cell development in the salivary gland
Source: eLife. 2017 Jun 17;6:e26620. doi: 10.7554/eLife.26620 (PMC5498133; doi:10.7554/eLife.26620)
Supplement: Figure 2—source data 3. — SMG+SLG from Krt14CreERT2; Rosa26mTmG and Krt14CreERT2; Rosa26mTmG; Sox2fl/fl were immunostained for SOX10 and AQP5 and GFP+ cells expressing SOX10 and AQP5 were quantified and expressed as a percentage of total positive cells. n = 3 glands/genotype and cells were counted in 3–4 acini/gland. s.d. = standard deviation. DOI: http://dx.doi.org/10.7554/eLife.26620.007 [file elife-26620-fig2-data3.docx]

**Figure 2 - source data 3.** Source data relating to Figure 2F. SMG+SLG from *Krt14^CreERT2^; Rosa26^mTmG^* and *Krt14^CreERT2^; Rosa26^mTmG^; Sox2^fl/fl^* were immunostained for SOX10 and AQP5 and GFP+ cells expressing SOX10 and AQP5 were quantified and expressed as a percentage of total positive cells. n = 3 glands/genotype and cells were counted in 3-4 acini/gland. s.d. = standard deviation.

|  | ***K14^CreERT2^;mTmG*** | s.d. | ***K14^CreERT2^;Sox2^flfl^;mTmG*** | s.d. |
| --- | --- | --- | --- | --- |
| AQP5+ | 92.54 | 4.07 | 5.22 | 2.15 |
| SOX10+ | 92.05 | 1.28 | 5.51 | 1.89 |
| KRT19+ | 98.38 | 1.48 | 96.01 | 2.29 |
