# Supplementary material for: SOX2 regulates acinar cell development in the salivary gland
Source: eLife. 2017 Jun 17;6:e26620. doi: 10.7554/eLife.26620 (PMC5498133; doi:10.7554/eLife.26620)
Supplement: Figure 2—source data 4. — qPCR for enrichment of Sox10 in SOX2 ChIP. n = 20 pooled SLG, average three experiments. s.d. = standard deviation. DOI: http://dx.doi.org/10.7554/eLife.26620.008 [file elife-26620-fig2-data4.docx]

**Figure 2 - source data 4.** Source data relating to Figure 2G. qPCR for enrichment of *Sox10* in SOX2 ChIP. n = 20 pooled SLG, average 3 experiments. s.d. = standard deviation.

|  | **Fold enrichment** | s.d. |
| --- | --- | --- |
| IgG | 0.83 | 0.09 |
| SOX2 | 2.92 | 0.83 |
