# Supplementary material for: SOX2 regulates acinar cell development in the salivary gland
Source: eLife. 2017 Jun 17;6:e26620. doi: 10.7554/eLife.26620 (PMC5498133; doi:10.7554/eLife.26620)
Supplement: Figure 2—figure supplement 1—source data 2. — Quantification of acini in Krt14CreERT2; Sox2fl/fl and wild-type (WT) glands at E16.5, with WT set to 100%. n = 3–7. s.d. = standard deviation. DOI: http://dx.doi.org/10.7554/eLife.26620.011 [file elife-26620-fig2-figsupp1-data2.docx]

**Figure 2 - Figure Supplement 1 – source data 2.** Source data relating to Figure 2 – Figure Supplement 1E. Quantification of acini in *Krt14^CreERT2^; Sox2^fl/fl^* and wild-type (WT) glands at E16.5, with WT set to 100%. n = 3-7. s.d. = standard deviation.

|  | **SMG** | s.d. | **SLG** | s.d. |
| --- | --- | --- | --- | --- |
| WT | 100.00 | 26.00 | 100.00 | 4.58 |
| *Krt14^CreERT2^;Sox2^fl/fl^* | 16.13 | 8.85 | 4.26 | 1.03 |
