# Supplementary material for: SOX2 regulates acinar cell development in the salivary gland
Source: eLife. 2017 Jun 17;6:e26620. doi: 10.7554/eLife.26620 (PMC5498133; doi:10.7554/eLife.26620)
Supplement: Figure 2—figure supplement 1—source data 3. — qPCR analysis of gene expression in Krt14CreERT2; Sox2fl/fl and wild-type (WT) glands at E13.5. Data were normalized to Rsp29 and WT. n = 3–4 SMG+SLG per genotype. s.d. = standard deviation. DOI: http://dx.doi.org/10.7554/eLife.26620.012 [file elife-26620-fig2-figsupp1-data3.docx]

**Figure 2 - Figure Supplement 1 – source data 3.** Source data relating to Figure 2 – Figure Supplement 1F. qPCR analysis of gene expression in *Krt14^CreERT2^; Sox2^fl/fl^* and wild-type (WT) glands at E13.5. Data were normalized to *Rsp29* and WT. n = 3-4 SMG+SLG per genotype. s.d. = standard deviation.

| **Gene** | **WT** | s.d. | ***Krt14^CreERT2^;Sox2^fl/fl^*** | s.d. |
| --- | --- | --- | --- | --- |
| *Trp53* | 1.00 | 0.13 | 1.93 | 0.52 |
| *Trp63* | 1.00 | 0.48 | 1.68 | 0.78 |
| *Ccnd1* | 1.00 | 0.19 | 1.67 | 0.35 |
| *Cdkn1a* | 1.00 | 0.07 | 1.49 | 0.37 |
| *Bax* | 1.00 | 0.16 | 1.92 | 0.47 |
| *Bbc3* | 1.00 | 0.46 | 3.09 | 0.89 |
| *Pmaip1* | 1.00 | 0.13 | 4.41 | 2.43 |
