# Supplementary material for: SOX2 regulates acinar cell development in the salivary gland
Source: eLife. 2017 Jun 17;6:e26620. doi: 10.7554/eLife.26620 (PMC5498133; doi:10.7554/eLife.26620)
Supplement: Figure 2—figure supplement 1—source data 4. — qPCR analysis of gene expression in Krt14CreERT2; Sox2fl/fl and wild-type (WT) glands at E16.5. Data were normalized to Rsp29 and WT. n = 3–4 SMG+SLG per genotype. s.d. = standard deviation. DOI: http://dx.doi.org/10.7554/eLife.26620.013 [file elife-26620-fig2-figsupp1-data4.docx]

**Figure 2 - Figure Supplement 1 – source data 4.** Source data relating to Figure 2 – Figure Supplement 1G. qPCR analysis of gene expression in *Krt14^CreERT2^; Sox2^fl/fl^* and wild-type (WT) glands at E16.5. Data were normalized to *Rsp29* and WT. n = 3-4 SMG+SLG per genotype. s.d. = standard deviation.

| **Gene** | **WT** | s.d. | ***Krt14^CreERT2^;Sox2^fl/fl^*** | s.d. |
| --- | --- | --- | --- | --- |
| *Ptch1* | 1.00 | 0.17 | 3.12 | 1.38 |
| *Zeb1* | 1.00 | 0.50 | 1.52 | 1.21 |
| *Zeb2* | 1.00 | 0.36 | 1.93 | 0.76 |
| *Dcpp2* | 1.00 | 0.65 | 0.87 | 1.47 |
| *Smgc* | 1.00 | 0.59 | 0.02 | 0.04 |
| *Pip* | 1.00 | 0.99 | 0.09 | 0.19 |
| *Spdef* | 1.00 | 0.09 | 0.24 | 0.24 |
| *Rab3d* | 1.00 | 1.26 | 0.22 | 0.23 |
| *Prol1* | 1.00 | 1.22 | 0.32 | 0.59 |
| *Trp53* | 1.00 | 0.41 | 0.78 | 0.48 |
| *dNp63* | 1.00 | 0.61 | 3.28 | 1.45 |
| *Ccnd1* | 1.00 | 0.37 | 0.63 | 0.58 |
| *Cdkn1a* | 1.00 | 0.42 | 10.72 | 6.07 |
| *Bax* | 1.00 | 0.12 | 2.54 | 1.32 |
| *Bbc3* | 1.00 | 0.30 | 5.33 | 3.27 |
| *Pmaip1* | 1.00 | 0.14 | 8.05 | 3.45 |
| *Etv4* | 1.00 | 0.21 | 2.52 | 0.68 |
| *Etv5* | 1.00 | 0.26 | 4.96 | 4.50 |
| *Fgf1* | 1.00 | 0.25 | 1.09 | 0.07 |
| *Fgf7* | 1.00 | 0.18 | 0.95 | 0.12 |
