# Supplementary material for: SOX2 regulates acinar cell development in the salivary gland
Source: eLife. 2017 Jun 17;6:e26620. doi: 10.7554/eLife.26620 (PMC5498133; doi:10.7554/eLife.26620)
Supplement: Figure 3—source data 1. — Quantification of the number of CASP3+ cells in acini of E11.5 Krt14CreERT2; Sox2fl/fl and wild-type (WT) glands cultured for 60 hr ± Z-VAD-FMK. n = 3 glands per treatment and cells were counted in 3–4 acini per gland. Data are the mean of three biological replicates and two experiments. s.d. = standard deviation. DOI: http://dx.doi.org/10.7554/eLife.26620.015 [file elife-26620-fig3-data1.docx]

**Figure 3 – source data 1.** Source data relating to Figure 3E. Quantification of the number of CASP3+ cells in acini of E11.5 *Krt14^CreERT2^; Sox2^fl/fl^* and wild-type (WT) glands cultured for 60h ± Z-VAD-FMK. n = 3 glands per treatment and cells were counted in 3-4 acini per gland. Data is the mean of 3 biological replicates and 2 experiments. s.d. = standard deviation.

|  | **WT** | s.d. | ***Krt14^CreERT2^;Sox2^fl/fl^*** | s.d. |
| --- | --- | --- | --- | --- |
| DMSO | 0.33 | 0.52 | 9.50 | 3.27 |
| +Z-VAD-FMK | 0.17 | 0.41 | 0.17 | 0.41 |
