# Supplementary material for: SOX2 regulates acinar cell development in the salivary gland
Source: eLife. 2017 Jun 17;6:e26620. doi: 10.7554/eLife.26620 (PMC5498133; doi:10.7554/eLife.26620)
Supplement: Figure 3—source data 2. — Quantification of the number of acini of E11.5 Krt14CreERT2; Sox2fl/fl and wild-type (WT) glands cultured for 60 hr ± Z-VAD-FMK. n = 3 glands per treatment. Data are means of three biological replicates and two experiments. s.d. = standard deviation. DOI: http://dx.doi.org/10.7554/eLife.26620.016 [file elife-26620-fig3-data2.docx]

**Figure 3 – source data 2.** Source data relating to Figure 3F. Quantification of the number of acini of E11.5 *Krt14^CreERT2^; Sox2^fl/fl^* and wild-type (WT) glands cultured for 60h ± Z-VAD-FMK. n = 3 glands per treatment. Data are means of 3 biological replicates and 2 experiments. s.d. = standard deviation.

|  | **WT** | s.d. | ***Krt14^CreERT2^;Sox2^fl/fl^*** | s.d. |
| --- | --- | --- | --- | --- |
| DMSO | 15.33 | 5.86 | 2.50 | 0.71 |
| +Z-VAD-FMK | 12.67 | 6.43 | 2.50 | 0.71 |
