# Supplementary material for: SOX2 regulates acinar cell development in the salivary gland
Source: eLife. 2017 Jun 17;6:e26620. doi: 10.7554/eLife.26620 (PMC5498133; doi:10.7554/eLife.26620)
Supplement: Figure 4—source data 1. — E13 murine SMG+SLG cultured for 48 hr ± parasympathetic ganglion (nerves). The number of acini were quantified. Data are means of three biological replicates and three experiments. s.d. = standard deviation. DOI: http://dx.doi.org/10.7554/eLife.26620.018 [file elife-26620-fig4-data1.docx]

**Figure 4 – source data 1.** Source data relating to Figure 4B. E13 murine SMG+SLG cultured for 48 h ± parasympathetic ganglion (nerves). The number of acini were quantified. Data are means of 3 biological replicates and 3 experiments. s.d. = standard deviation.

|  | **SMG** | s.d. | **SLG** | s.d. |
| --- | --- | --- | --- | --- |
| + nerves | 26.25 | 1.00 | 12.00 | 1.15 |
| - nerves | 8.50 | 4.27 | 5.00 | 1.41 |
