# Supplementary material for: SOX2 regulates acinar cell development in the salivary gland
Source: eLife. 2017 Jun 17;6:e26620. doi: 10.7554/eLife.26620 (PMC5498133; doi:10.7554/eLife.26620)
Supplement: Figure 4—source data 2. — E13 murine SMG+SLG cultured for 48 hr ± parasympathetic ganglion (nerves) and subjected to immunofluorescent analysis. The number of AQP5+ and SOX10+ cells were quantified. Data are means of three biological replicates and three experiments. s.d. = standard deviation. DOI: http://dx.doi.org/10.7554/eLife.26620.019 [file elife-26620-fig4-data2.docx]

**Figure 4 – source data 2.** Source data relating to Figure 4C. E13 murine SMG+SLG cultured for 48 h ± parasympathetic ganglion (nerves) and subjected to immunofluorescent analysis. The number of AQP5+ and SOX10+ cells were quantified. Data are means of 3 biological replicates and 3 experiments. s.d. = standard deviation.

|  | **AQP5+** | s.d. | **SOX10+** | s.d. |
| --- | --- | --- | --- | --- |
| + nerves | 43.00 | 14.38 | 46.57 | 10.88 |
| - nerves | 8.83 | 4.31 | 16.86 | 8.86 |
