# Supplementary material for: SOX2 regulates acinar cell development in the salivary gland
Source: eLife. 2017 Jun 17;6:e26620. doi: 10.7554/eLife.26620 (PMC5498133; doi:10.7554/eLife.26620)
Supplement: Figure 4—source data 3. — E11.5 murine SMG+SLG deficient in Phox2b were cultured for 60 hr. The number of acini were quantified. Data are means of three biological replicates and three experiments. s.d. = standard deviation. DOI: http://dx.doi.org/10.7554/eLife.26620.020 [file elife-26620-fig4-data3.docx]

**Figure 4 – source data 3.** Source data relating to Figure 4E. E11.5 murine SMG+SLG deficient in *Phox2b* were cultured for 60 h. The number of acini were quantified. Data are means of 3 biological replicates and 3 experiments. s.d. = standard deviation.

|  | **SMG** | s.d. | **SLG** | s.d. |
| --- | --- | --- | --- | --- |
| WT | 11.25 | 1.71 | 2.75 | 0.96 |
| *Phox2b^LacZ/LacZ^* | 1.75 | 0.50 | 1.00 | 0.00 |
