# Supplementary material for: SOX2 regulates acinar cell development in the salivary gland
Source: eLife. 2017 Jun 17;6:e26620. doi: 10.7554/eLife.26620 (PMC5498133; doi:10.7554/eLife.26620)
Supplement: Figure 4—source data 4. — E11.5 murine SMG+SLG deficient in Phox2b were cultured for 60 hr and qPCR performed. Data were normalized to Rsp29 and the WT. Data are means of three biological replicates and three experiments. s.d. = standard deviation. DOI: http://dx.doi.org/10.7554/eLife.26620.021 [file elife-26620-fig4-data4.docx]

**Figure 4 – source data 4.** Source data relating to Figure 4F. E11.5 murine SMG+SLG deficient in *Phox2b* were cultured for 60 h and qPCR performed. Data were normalized to *Rsp29* and the WT. Data are means of 3 biological replicates and 3 experiments. s.d. = standard deviation.

| **Gene** | **WT** | s.d. | ***Phox2b^LacZ/LacZ^*** | s.d. |
| --- | --- | --- | --- | --- |
| *Cdh1* | 1.00 | 0.23 | 0.70 | 0.26 |
| *Tubb3* | 1.00 | 0.48 | 0.01 | 0.01 |
| *Vip* | 1.00 | 0.55 | 0.00 | 0.00 |
| *Vacht* | 1.00 | 0.66 | 0.04 | 0.03 |
| *Syn2* | 1.00 | 0.53 | 0.03 | 0.01 |
| *Chrm1* | 1.00 | 0.76 | 0.21 | 0.30 |
| *Krt5* | 1.00 | 1.10 | 2.32 | 1.37 |
| *Sox2* | 1.00 | 0.90 | 0.58 | 0.33 |
| *Aqp5* | 1.00 | 0.59 | 0.24 | 0.34 |
| *Chrm3* | 1.00 | 0.88 | 0.61 | 0.22 |
| *Mist1* | 1.00 | 0.47 | 0.88 | 0.35 |
| *Sox10* | 1.00 | 0.62 | 0.24 | 0.23 |
| *Krt7* | 1.00 | 0.55 | 0.91 | 0.24 |
| *Krt19* | 1.00 | 0.53 | 1.61 | 0.08 |
| *Egfr* | 1.00 | 0.48 | 0.82 | 0.53 |
| *Fgfr2b* | 1.00 | 1.01 | 0.84 | 0.20 |
