# Supplementary material for: SOX2 regulates acinar cell development in the salivary gland
Source: eLife. 2017 Jun 17;6:e26620. doi: 10.7554/eLife.26620 (PMC5498133; doi:10.7554/eLife.26620)
Supplement: Figure 5—source data 1. — E14 mouse SLG epithelia cultured with FGF10 ±CCh for 24 hr. The number of SOX2+, EdU+ and SOX2+EdU+ cells were quantified. Data are means of three biological replicates and three experiments. s.d. = standard deviation. DOI: http://dx.doi.org/10.7554/eLife.26620.023 [file elife-26620-fig5-data1.docx]

**Figure 5 – source data 1.** Source data relating to Figure 5B. E14 mouse SLG epithelia cultured with FGF10 ±CCh for 24 h. The number of SOX2+, EdU+ and SOX2+EdU+ cells were quantified. Data are means of 3 biological replicates and 3 experiments. s.d. = standard deviation.

|  | **SOX2+** | s.d. | **EdU+** | s.d. | **SOX2+EdU+** | s.d. |
| --- | --- | --- | --- | --- | --- | --- |
| -CCh | 9.01 | 8.35 | 11.79 | 8.25 | 1.31 | 1.63 |
| +CCh | 34.23 | 13.48 | 26.58 | 6.02 | 6.92 | 4.70 |
