# Supplementary material for: SOX2 regulates acinar cell development in the salivary gland
Source: eLife. 2017 Jun 17;6:e26620. doi: 10.7554/eLife.26620 (PMC5498133; doi:10.7554/eLife.26620)
Supplement: Figure 5—source data 2. — E14 mouse SLG cultured for 24 hr with DMSO or 4-DAMP (10 µM). The number of SOX2+ and SOX2+Ki67+ cells were counted via FACS, normalized to control and expressed as percentage of total ECAD+ cells. s.d. = standard deviation. DOI: http://dx.doi.org/10.7554/eLife.26620.024 [file elife-26620-fig5-data2.docx]

**Figure 5 – source data 2.** Source data relating to Figure 5C. E14 mouse SLG cultured for 24 h with DMSO or 4-DAMP (10 µM). The number of SOX2+ and SOX2+Ki67+ cells were counted via FACS, normalised to control and expressed as percentage of total ECAD+ cells. s.d. = standard deviation.

|  | **SOX2+** | s.d. | **SOX2+Ki67+** | s.d. |
| --- | --- | --- | --- | --- |
| DMSO | 12.59 | 1.56 | 5.85 | 0.46 |
| +4-DAMP | 3.53 | 0.48 | 2.61 | 0.78 |
