# Supplementary material for: SOX2 regulates acinar cell development in the salivary gland
Source: eLife. 2017 Jun 17;6:e26620. doi: 10.7554/eLife.26620 (PMC5498133; doi:10.7554/eLife.26620)
Supplement: Figure 5—source data 3. — E13 SMG+SLG were cultured ± ganglia and ± CCh (100 nM) for 48 hr and the number of AQP5+ and KRT19+ cells counted. Counts were normalized to the control (nerves). Data are means of three biological replicates and three experiments. s.d. = standard deviation. DOI: http://dx.doi.org/10.7554/eLife.26620.025 [file elife-26620-fig5-data3.docx]

**Figure 5 – source data 3.** Source data relating to Figure 5G. E13 SMG+SLG were cultured ± ganglia and ± CCh (100 nM) for 48 h and the number of AQP5+ and KRT19+ cells counted. Counts were normalized to the control (nerves). Data are means of 3 biological replicates and 3 experiments. s.d. = standard deviation.

|  | **AQP5+** | s.d. | **KRT19+** | s.d. |
| --- | --- | --- | --- | --- |
| Nerves | 100.00 | 27.45 | 100.00 | 20.16 |
| No nerves | 26.79 | 8.51 | 110.74 | 15.89 |
| No nerves+CCh | 91.34 | 33.98 | 97.09 | 16.32 |
