# Supplementary material for: SOX2 regulates acinar cell development in the salivary gland
Source: eLife. 2017 Jun 17;6:e26620. doi: 10.7554/eLife.26620 (PMC5498133; doi:10.7554/eLife.26620)
Supplement: Figure 5—source data 4. — E13 SMG+SLG were cultured ± ganglia and ± CCh (100 nM) for 48 hr and subjected to qPCR analysis. Data were normalized to Rsp29 and control (nerves). Data are means of three biological replicates and three experiments. s.d. = standard deviation. DOI: http://dx.doi.org/10.7554/eLife.26620.026 [file elife-26620-fig5-data4.docx]

**Figure 5 – source data 4.** Source data relating to Figure 5H. E13 SMG+SLG were cultured ± ganglia and ± CCh (100 nM) for 48 h and subjected to qPCR analysis. Data were normalized to *Rsp29* and control (nerves). Data are means of 3 biological replicates and 3 experiments. s.d. = standard deviation.

| **Gene** | **Nerves** | s.d. | **No nerves** | s.d. | **No nerves+CCh** | s.d. |
| --- | --- | --- | --- | --- | --- | --- |
| *Cdh1* | 1.00 | 0.04 | 0.77 | 0.09 | 0.76 | 0.17 |
| *Tubb3* | 1.00 | 0.22 | 0.02 | 0.00 | 0.02 | 0.01 |
| *Vip* | 1.00 | 0.14 | 0.04 | 0.03 | 0.01 | 0.01 |
| *Vacht* | 1.00 | 0.50 | 0.00 | 0.00 | 0.00 | 0.00 |
| *Syn2* | 1.00 | 0.25 | 0.10 | 0.09 | 0.04 | 0.02 |
| *Chrm1* | 1.00 | 1.13 | 0.95 | 0.40 | 1.58 | 0.61 |
| *Krt5* | 1.00 | 0.19 | 0.84 | 0.16 | 1.39 | 0.45 |
| *Sox2* | 1.00 | 0.20 | 0.55 | 0.08 | 0.82 | 0.17 |
| *Aqp5* | 1.00 | 0.11 | 0.40 | 0.09 | 0.74 | 0.17 |
| *Chrm3* | 1.00 | 0.22 | 0.78 | 0.14 | 1.37 | 0.60 |
| *Mist1* | 1.00 | 0.07 | 0.52 | 0.23 | 0.93 | 0.23 |
| *Sox10* | 1.00 | 0.13 | 0.51 | 0.11 | 1.19 | 0.27 |
| *Krt7* | 1.00 | 0.18 | 1.34 | 0.40 | 1.20 | 0.29 |
| *Krt19* | 1.00 | 0.10 | 1.27 | 0.12 | 1.62 | 0.38 |
| *Egfr* | 1.00 | 0.33 | 0.90 | 0.19 | 1.10 | 0.23 |
| *Fgfr2b* | 1.00 | 0.22 | 0.96 | 0.16 | 1.26 | 0.28 |
