# Supplementary material for: SOX2 regulates acinar cell development in the salivary gland
Source: eLife. 2017 Jun 17;6:e26620. doi: 10.7554/eLife.26620 (PMC5498133; doi:10.7554/eLife.26620)
Supplement: Figure 5—figure supplement 1—source data 1. — E14 mouse SLGs cultured for 4 hr with DMSO or the muscarinic inhibitor 4-DAMP (10 µM) and subjected to gene profiling by qPCR. Data were normalized to Rsp29 and control values (DMSO). s.d. = standard deviation. DOI: http://dx.doi.org/10.7554/eLife.26620.028 [file elife-26620-fig5-figsupp1-data1.docx]

**Figure 5 - Figure Supplement 1 – source data 1.** Source data relating to Figure 5 – Figure Supplement 1C. E14 mouse SLGs cultured for 4h with DMSO or the muscarinic inhibitor 4-DAMP (10 µM) and subjected to gene profiling by qPCR. Data were normalized to *Rsp29* and control values (DMSO). s.d. = standard deviation.

|  | **DMSO** | s.d. | **+4-DAMP** | s.d. |
| --- | --- | --- | --- | --- |
| *Cdh1* | 1.00 | 0.04 | 0.94 | 0.06 |
| *Sox2* | 1.00 | 0.06 | 0.38 | 0.04 |
| *Krt5* | 1.00 | 0.16 | 0.55 | 0.27 |
| *Aqp5* | 1.00 | 0.11 | 0.52 | 0.13 |
| *Chrm3* | 1.00 | 0.09 | 0.21 | 0.02 |
| *Mist1* | 1.00 | 0.03 | 0.55 | 0.03 |
| *Sox10* | 1.00 | 0.08 | 0.80 | 0.07 |
| *Krt7* | 1.00 | 0.07 | 0.99 | 0.02 |
| *Krt19* | 1.00 | 0.04 | 1.89 | 0.30 |
| *Egfr* | 1.00 | 0.13 | 1.68 | 0.17 |
| *Tubb3* | 1.00 | 0.09 | 1.08 | 0.28 |
| *Vip* | 1.00 | 0.15 | 1.08 | 0.04 |
| *Chrm1* | 1.00 | 0.11 | 0.34 | 0.05 |
