# Supplementary material for: SOX2 regulates acinar cell development in the salivary gland
Source: eLife. 2017 Jun 17;6:e26620. doi: 10.7554/eLife.26620 (PMC5498133; doi:10.7554/eLife.26620)
Supplement: Figure 5—figure supplement 1—source data 2. — E14 mouse SLGs cultured for 24 hr with DMSO or the muscarinic inhibitor 4-DAMP (10 µM). The number of AQP5+, KRT19+, SOX10+, Ki67+ remaining in SLG cultured with or without 4-DAMP for 24 hr were quantified. n = 2 SLG per treatment and cells were counted in 3–4 end acini per gland. s.d. = standard deviation. DOI: http://dx.doi.org/10.7554/eLife.26620.029 [file elife-26620-fig5-figsupp1-data2.docx]

**Figure 5 - Figure Supplement 1 – source data 2.** Source data relating to Figure 5 – Figure Supplement 1E. E14 mouse SLGs cultured for 24 h with DMSO or the muscarinic inhibitor 4-DAMP (10 µM). The number of AQP5+, KRT19+, SOX10+, Ki67+ remaining in SLG cultured with or without 4-DAMP for 24h were quantified. n = 2 SLG per treatment and cells were counted in 3-4 end acini per gland. s.d. = standard deviation.

|  | **DMSO** | s.d. | **+4-DAMP** | s.d. |
| --- | --- | --- | --- | --- |
| AQP5 | 100.00 | 14.20 | 40.23 | 1.95 |
| KRT19 | 100.00 | 26.38 | 110.91 | 33.98 |
| SOX10 | 100.00 | 32.45 | 41.10 | 18.52 |
| Ki67 | 100.00 | 18.92 | 38.31 | 4.16 |
