# Supplementary material for: SOX2 regulates acinar cell development in the salivary gland
Source: eLife. 2017 Jun 17;6:e26620. doi: 10.7554/eLife.26620 (PMC5498133; doi:10.7554/eLife.26620)
Supplement: Figure 5—figure supplement 1—source data 3. — E14 mouse SLGs cultured for 4 hr with PD 168393 (20 μM), KN-93 (15 μM) or vehicle (water; Control) were subjected to gene profiling by qPCR. Data were normalized to Rsp29 and control values. Data are means of three to four SGs per treatment/genotype. s.d. = standard deviation. DOI: http://dx.doi.org/10.7554/eLife.26620.030 [file elife-26620-fig5-figsupp1-data3.docx]

**Figure 5 - Figure Supplement 1 – source data 3.** Source data relating to Figure 5 – Figure Supplement 1F. E14 mouse SLGs cultured for 4h with PD 168393 (20 μM), KN-93 (15 μM) or vehicle (water; Control) were subjected to gene profiling by qPCR. Data were normalized to *Rsp29* and control values. Data are means of 3-4 SGs per treatment/genotype. s.d. = standard deviation.

|  | **Control** | s.d. | **+PD 168393** | s.d. | **+KN-93** | s.d. |
| --- | --- | --- | --- | --- | --- | --- |
| *Cdh1* | 1.00 | 0.02 | 1.57 | 0.21 | 0.92 | 0.03 |
| *Sox2* | 1.00 | 0.08 | 0.99 | 0.15 | 0.56 | 0.02 |
| *Krt5* | 1.00 | 0.23 | 0.50 | 0.07 | 0.82 | 0.05 |
| *Aqp5* | 1.00 | 0.09 | 0.76 | 0.10 | 0.43 | 0.12 |
| *Chrm3* | 1.00 | 0.11 | 0.41 | 0.09 | 0.42 | 0.11 |
| *Mist1* | 1.00 | 0.11 | 0.69 | 0.10 | 0.65 | 0.08 |
| *Sox10* | 1.00 | 0.05 | 1.00 | 0.16 | 0.66 | 0.03 |
| *Krt19* | 1.00 | 0.07 | 0.80 | 0.07 | 1.03 | 0.13 |
| *Egfr* | 1.00 | 0.28 | 3.71 | 0.26 | 2.15 | 0.18 |
| *Tubb3* | 1.00 | 0.13 | 3.31 | 0.49 | 2.35 | 0.15 |
| *Vip* | 1.00 | 0.12 | 3.12 | 0.60 | 0.99 | 0.29 |
| *Chrm1* | 1.00 | 0.20 | 0.42 | 0.17 | 0.68 | 0.23 |
