# Supplementary material for: SOX2 regulates acinar cell development in the salivary gland
Source: eLife. 2017 Jun 17;6:e26620. doi: 10.7554/eLife.26620 (PMC5498133; doi:10.7554/eLife.26620)
Supplement: Figure 6—source data 1. — Human fetal SLG explants cultured with murine E13 PSG or mesenchyme for 7 days were subjected to gene profiling by qPCR. Data were normalized to GAPDH and control (+ mesenchyme). Data are means of six biological replicates, two individual experiments. s.d. = standard deviation. DOI: http://dx.doi.org/10.7554/eLife.26620.032 [file elife-26620-fig6-data1.docx]

**Figure 6 – source data 1.** Source data relating to Figure 6E. Human fetal SLG explants cultured with murine E13 PSG or mesenchyme for 7 days were subjected to gene profiling by qPCR. Data were normalized to *GAPDH* and control (+ mesenchyme*)*. Data are means of 6 biological replicates, 2 individual experiments. s.d. = standard deviation.

| **Gene** | **20w hSLG** | s.d. | **22w hSLG** | s.d. |
| --- | --- | --- | --- | --- |
| *CDH1* | 1.14 | 0.10 | 1.33 | 0.04 |
| *SOX2* | 7.20 | 1.58 | 3.05 | 0.20 |
| *AQP3* | 1.56 | 0.43 | 1.85 | 0.15 |
| *MIST1* | 5.19 | 1.07 | 6.66 | 1.36 |
| *CHRM1* | 9.83 | 2.60 | 2.01 | 0.54 |
| *CHRM3* | 2.59 | 0.34 | 3.42 | 0.16 |
| *SOX10* | 0.86 | 0.06 | 0.93 | 0.08 |
| *CD44* | 1.33 | 0.18 | 0.96 | 0.06 |
| *KRT5* | 0.87 | 0.12 | 2.10 | 0.12 |
| *KRT7* | 0.97 | 0.10 | 0.87 | 0.16 |
| *KRT8* | 1.30 | 0.20 | 1.06 | 0.04 |
| *KRT14* | 0.90 | 0.15 | 1.46 | 0.19 |
| *KRT19* | 1.36 | 0.22 | 1.98 | 0.16 |
| *KIT* | 0.98 | 0.11 | 1.65 | 0.08 |
| *EGFR* | 0.70 | 0.07 | 1.72 | 0.23 |
