# Supplementary material for: SOX2 regulates acinar cell development in the salivary gland
Source: eLife. 2017 Jun 17;6:e26620. doi: 10.7554/eLife.26620 (PMC5498133; doi:10.7554/eLife.26620)
Supplement: Figure 6—source data 2. — qPCR analysis of fetal human SLG (22–23 w) dissociated cells cultured ± CCh for 48 hr. Data were normalized to GAPDH and control (-CCh). Data are means of six biological replicates, two individual experiments. s.d. = standard deviation. DOI: http://dx.doi.org/10.7554/eLife.26620.033 [file elife-26620-fig6-data2.docx]

**Figure 6 – source data 2.** Source data relating to Figure 6F. qPCR analysis of fetal human SLG (22-23 w) dissociated cells cultured ± CCh for 48h. Data were normalized to *GAPDH* and control (-CCh). Data are means of 6 biological replicates, 2 individual experiments. s.d. = standard deviation.

| **Gene** | **-CCh** | s.d. | **+CCh** | s.d. |
| --- | --- | --- | --- | --- |
| *CDH1* | 1.00 | 0.21 | 5.38 | 2.44 |
| *SOX2* | 1.00 | 0.14 | 7.18 | 1.18 |
| *SOX10* | 1.00 | 0.25 | 0.04 | 0.01 |
| *CHRM3* | 1.00 | 0.00 | 22.05 | 12.13 |
| *CHRM1* | 1.00 | 0.01 | 10.06 | 1.65 |
| *AQP3* | 1.00 | 0.36 | 9.35 | 1.71 |
| *MIST1* | 1.00 | 0.14 | 7.19 | 2.97 |
| *CD44* | 1.00 | 0.30 | 8.28 | 1.91 |
| *KRT5* | 1.00 | 0.07 | 35.20 | 4.69 |
| *KRT7* | 1.00 | 0.12 | 1.14 | 0.21 |
| *KRT14* | 1.00 | 0.14 | 1.30 | 0.14 |
| *EGFR* | 1.00 | 0.11 | 2.27 | 0.31 |
| *KIT* | 1.00 | 0.08 | 2.34 | 0.26 |
