# Supplementary material for: SOX2 regulates acinar cell development in the salivary gland
Source: eLife. 2017 Jun 17;6:e26620. doi: 10.7554/eLife.26620 (PMC5498133; doi:10.7554/eLife.26620)
Supplement: Figure 6—source data 3. — qPCR analysis of fetal human SLG (22–23 w) explants cultured ± CCh for 72 hr. Data were normalized to GAPDH and control (-CCh). Data are means of six biological replicates, two individual experiments. s.d. = standard deviation. DOI: http://dx.doi.org/10.7554/eLife.26620.034 [file elife-26620-fig6-data3.docx]

**Figure 6 – source data 3.** Source data relating to Figure 6G. qPCR analysis of fetal human SLG (22-23 w) explants cultured ± CCh for 72h. Data were normalized to *GAPDH* and control (-CCh). Data are means of 6 biological replicates, 2 individual experiments. s.d. = standard deviation.

| **Gene** | **-CCh** | s.d. | **+CCh** | s.d. |
| --- | --- | --- | --- | --- |
| *CDH1* | 1.00 | 0.00 | 1.27 | 0.08 |
| *SOX2* | 1.00 | 0.65 | 2.99 | 0.42 |
| *SOX10* | 1.00 | 0.00 | 1.90 | 0.20 |
| *CHRM3* | 1.00 | 0.15 | 1.09 | 0.12 |
| *CHRM1* | 1.00 | 0.17 | 3.99 | 0.14 |
| *AQP3* | 1.00 | 0.00 | 1.39 | 0.07 |
| *MIST1* | 1.00 | 0.16 | 0.97 | 0.13 |
| *CD44* | 1.00 | 0.21 | 2.70 | 0.55 |
| *KRT5* | 1.00 | 0.24 | 1.78 | 0.07 |
| *KRT7* | 1.00 | 0.19 | 1.00 | 0.35 |
| *KRT14* | 1.00 | 0.10 | 0.75 | 0.09 |
| *EGFR* | 1.00 | 0.12 | 0.48 | 0.30 |
| *KIT* | 1.00 | 0.13 | 0.92 | 0.10 |
