# Supplementary material for: SOX2 regulates acinar cell development in the salivary gland
Source: eLife. 2017 Jun 17;6:e26620. doi: 10.7554/eLife.26620 (PMC5498133; doi:10.7554/eLife.26620)
Supplement: Figure 6—source data 4. — Analysis of fetal human SLG (22–23 w) dissociated cells cultured ± CCh for 48 hr. The number of ECAD+SOX2+ and ECAD+SOX2+Ki67+ cells were measured by FACS as a percentage of total ECAD+ cells. Each # represents an independent experiment. s.d. = standard deviation. DOI: http://dx.doi.org/10.7554/eLife.26620.035 [file elife-26620-fig6-data4.docx]

**Figure 6 – source data 4.** Source data relating to Figure 6H. Analysis of fetal human SLG (22-23 w) dissociated cells cultured ± CCh for 48h. The number of ECAD+SOX2+ and ECAD+SOX2+Ki67+ cells were measured by FACS as a percentage of total ECAD+ cells. Each # represents an independent experiment. s.d. = standard deviation.

|  | **SOX2+ cells** | | **SOX2+EdU+ cells** | |
| --- | --- | --- | --- | --- |
|  | **Control** | **+CCh** | **Control** | **+CCh** |
| #1 | 21.40 | 27.68 | 4.70 | 7.78 |
| #2 | 12.18 | 17.81 | 2.28 | 3.61 |
| #3 | 11.74 | 13.71 | 2.20 | 4.42 |
